# Chondromatosis of the Lumbar Spine: Minimally Invasive Spine Surgery for This Rare Condition

**DOI:** 10.7759/cureus.71231

**Published:** 2024-10-10

**Authors:** Marcos G Baabor, Bayron Valenzuela Cecchi, Adrian Abrego, Pedro Vázquez, Patricia Orellana, Facundo Las Heras

**Affiliations:** 1 Department of Neurology and Neurosurgery, Hospital Clínico de la Universidad de Chile, Santiago, CHL; 2 Department of Neurosurgery, Hospital General La Villa, Secretaria de Salud CDMX, Ciudad de Mexico, MEX; 3 Department of Radiology, Hospital Clínico de la Universidad de Chile, Santiago, CHL; 4 Department of Pathology, Hospital Clínico de la Universidad de Chile, Santiago, CHL

**Keywords:** lateral transpsoas approach, lower back pain (lbp), lumbar spine surgery, minimally invasive spine surgery, synovial chondromatosis

## Abstract

Synovial chondromatosis (SC) is a rare, benign disease. It usually occurs in large joints such as the hip and knee. Few cases have been reported in the spine, especially in the lumbar spine. It is characterized by the presence of clusters of chondrocytes within the joints and free in the joint cavity. The main symptom is pain. Diagnosis requires a high level of suspicion, and malignancy must always be ruled out. Surgical management is a challenge. We present the case of a patient with an extensive spinal tumor that was managed under the principles of minimal spinal invasion. The exeresis was performed in three different surgical times, achieving the total exeresis of the tumor with low morbidity.

## Introduction

Synovial chondromatosis (SC) is a rare, benign condition. Although initially classified as cartilaginous metaplasia, more recently, SC has been shown to be a neoplastic process, characterized by islets of chondrocytes within the synovial joints, resulting in thickening of the synovial tissue with free subsynovial chondroid nodules in the joint cavity. 

This disease may also be known as synovial osteochondromatosis, synovial chondrometaplasia, articular echondrosis, and synovial chondrosis [1-3].

Cartilaginous nodules may protrude from the synovial tissue, divide, and calcify, with the characteristic imaging result of multiple calcifications and bony erosive phenomena on the CT scan. Synovial chondromatosis usually affects the large appendicular joints, most commonly the hip and knee, and rarely the spine [4-8].

The free calcified fragments are responsible for the symptomatology due to the compression of neural structures. The pain may be nonspecific, as it can be at rest or in movement. These debris are similar to those observed in an inflammatory process [3, 9].

Synovial chondromatosis can be classified into primary and secondary. Primary SC is a neoplastic process with exuberant subsynovial hyaline cartilage growth, typically in the absence of any underlying arthritic facet disease [4]. Secondary SC can be caused by other joint diseases such as degenerative osteoarthritis, osteonecrosis, traumatic injury, neuropathic osteoarthropathy, osteochondral fractures, tuberculosis, and rheumatoid arthritis [10].

We present the case of a large SC of the lumbar spine, which was managed in a multi-stage approach with a minimally invasive spine technique with fewer risks, short hospital stays, and good clinical outcomes [11].

## Case presentation

We present the case of a 55-year-old male patient, a driver and marathon runner, with the only history of renal lithiasis treated 20 years ago.

The patient consulted in our institution for right radicular back pain of six months of evolution with regular response to medical treatment. On neurological examination, no motor or sensory alterations. There were only signs of radicular irritation (Lasegue`s sign).

**Figure 1:**
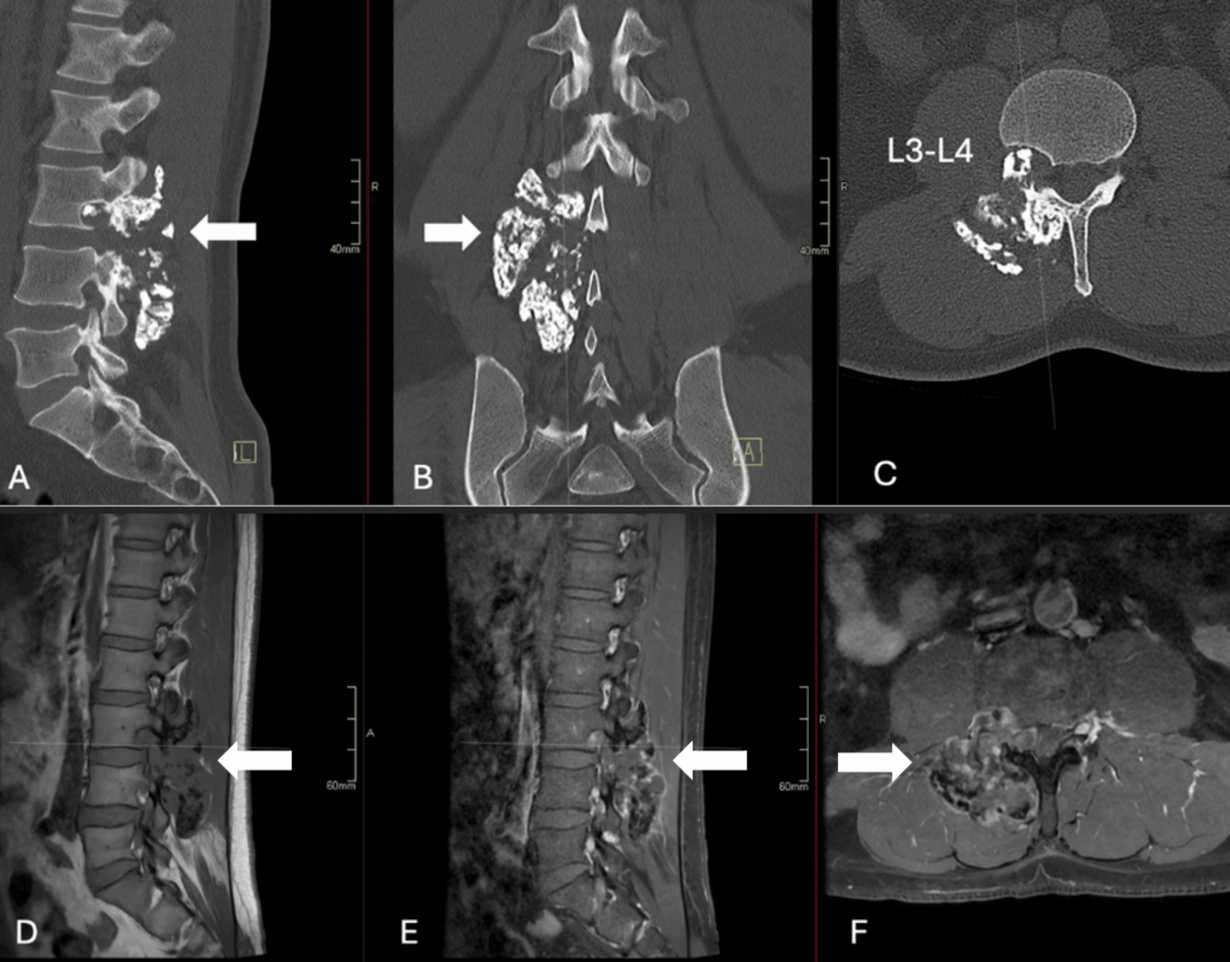
CT scans (sagittal (A), coronal (B), and axial (C) views); MRI (sagittal T1 (D), sagittal T1 Gd (E), and axial T1 Gd (F) views) The CT scan shows a right lumbar paraspinal mass between L3 and L5 with multiple clustered chondral calcifications and erosive involvement of the facet joints and L3 cortical rim. It occupies the L3-L4 foraminal canal (arrow in A and B). On MRI, it is seen as a polybulinous mass with heterogeneous signals on T1 and T2 with scant gadolinium enhancement. At L3, it shows slight compression of the dural sac to the left. Gd: gadolinium

Computed tomography showed multiple calcified nodules in relation to the facet joint L3-L4. Complementary MRI showed a heterogeneous right lumbar paraspinal mass of 9 cm craniocaudal, 6 cm in the anteroposterior axis, and 6 cm transverse axis, with erosion of the right facet joints of L2-L3 and L4-L5, but with destruction of the ipsilateral facet joint of L3-L4. The margins of the lesion were irregular, with the mass insinuating into the right neuroforamina of L3-L4 with compression of the neural structures. The lesion was predominantly isointense on T1, with scattered hypointense areas. On T2, the predominant signal was hyperintense with scattered hypointense areas. Gadolinium showed areas of heterogeneous enhancement, predominantly angular (Figure 1).

It was decided to perform a biopsy by interventional radiology technique to determine the histopathology of the tumor lesion. The biopsy showed hyaline cartilage with small, mature chondrocytes and the presence of calcifications suggestive of SC.

**Figure 2:**
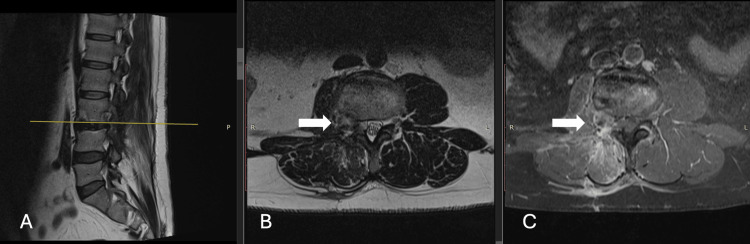
Sagittal (A), axial (B) and axial T1 Gd (C) MRI views Images after the second surgery show the L3-L4 intersomatic cage (lateral lumbar interbody fusion (LLIF)) and preserved facet joint with the tumor. No paraspinal tumor is observed. Gd: gadolinium

**Figure 3:**
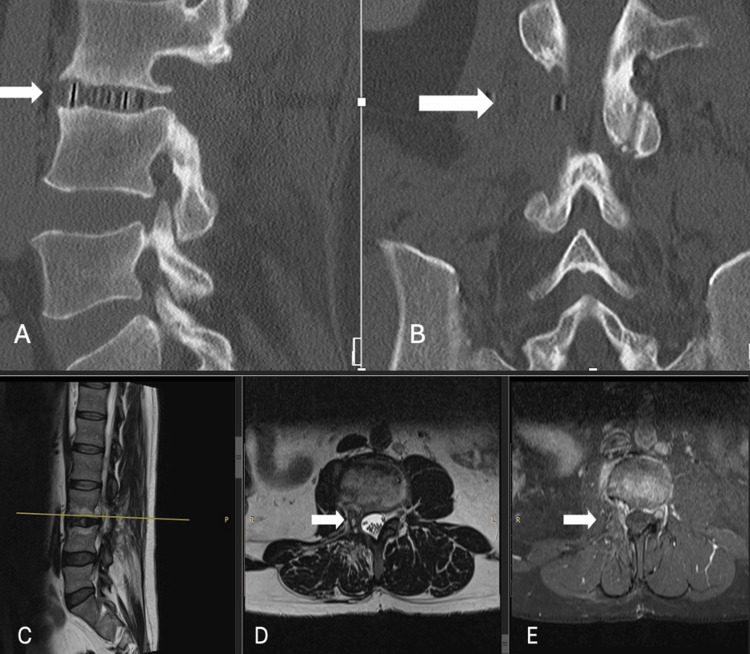
CT scans (sagittal (A) and coronal (B) views); MRI (sagittal (C),axial T2 (D), and axial T1 Gd (E) Post-surgical changes with L3-L4 intersomatic cage (lateral lumbar interbody fusion (LLIF)) and resection of the right L3-L4 facet joint (arrow in A and B). The MRI shows gadolinium (Gd)-enhancing scar tissue in the right perivertebral location, in the ipsilateral foraminal and paraspinous canals (arrows in D and E).

For this unusual case, we decided to perform three minimally invasive surgeries at different times in order to maintain functionality and the patient's wishes (marathon running). The first surgery was performed to resect the right foraminal and extraforaminal components, leaving the component adjacent to the L3-L4 facet joint (November 2022). In a second surgical stage, a lateral interbody fusion was performed using a transpsoas approach (stand-alone lateral lumbar interbody fusion (LLIF)) with an interbody fusion cage due to the imminent risk of instability (December 2022) (Figure 2). With the fusion completed, six months later the final component resection was performed, with L3-L4 facetectomy, partial resection of the vertebral body of L3, and hemilaminectomy of L3-L4, resulting in a total resection (April 2023) (Figure 3). All procedures with minimal bleeding, with a one-day-to-two-day hospital stay per procedure, allowing the patient to return to his activities as soon as possible. Each procedure was performed without requiring the patient to take time away from work.

## Discussion

Synovial chondromatosis is a rare disease with an unknown cause. It is a local, aggressive, but benign tumor process characterized by the formation of multiple cartilaginous nodules adjacent to a synovial joint. Synovial chondromatosis usually affects the large appendicular joints, most commonly the hip and knee, and rarely the spine [1, 3].

Although much has been written about SC in appendicular joints, there are few reported cases in the spine in the literature. The official number is unknown, but probably fewer than 50 cases have been reported in the literature. Shaw et al. published case number 10 in 2014 [5, 7]. Involvement of the spine is very rare, with the cervical spine being the most affected area. Littrell et al. (2016) published a series on 28 patients; 16 (55%) were located in the cervical spine, six (20%) thoracic, six (20%) lumbar, and one (3%) in the sacrum [1]. The average lesion size was 3.4 cm, with a range of 1.6 to 6 cm. Twenty-two cases (79%) had an epidural component, 18 (64%) had a foraminal component, and 16 (57%) had a paraspinal component.

Synovial chondromatosis typically presents as an epidural soft tissue mass with neuroforaminal extension, in addition to posterior paraspinal involvement. In relation to the articular facet, Littrel et al. [1] found in their series that the lesion was found very close to the articular facet in 96% of cases, however, without finding clear evidence of facet invasion. One theory for the lack of destruction is the small size and close relationship of their joints, which allows the tissue to expand into areas of lesser resistance.

Synovial chondromatosis mainly affects patients between the third and fifth decade of life, with a male predominance. It is estimated that there is a male-to-female ratio of 2:1. A mean age of 41 years (22-68 years) has been reported [7].

Approximately 6.4% of cases may have a malignant transformation to chondrosarcoma. It should be noted that diagnosis is challenging, as radiological and imaging findings can be very similar to the classic findings of SC. In a systematic review of chondrosarcoma, the transformation time interval was approximately 10 years [12].

**Figure 4:**
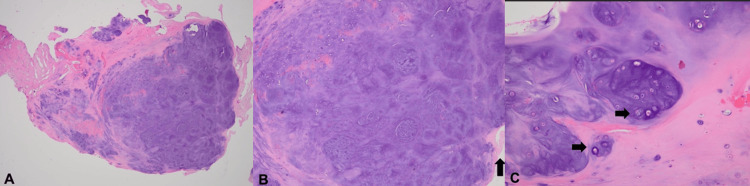
Histopathology images of the patient A) Hyaline cartilage mass is arranged in a nodular growth pattern (H&E, ×20); (B) nodular proliferation of the hyaline cartilage adjacent to synovial tissue is visualized (arrow; H&E, x40); (C) the chondrocytes are clustered with different sizes of nuclei but without atypia (arrow; H&E, ×200).

Regarding histological findings, multiple nodules of hyaline cartilage are observed in relation to the synovium or free in the joint space. Microscopically, multiple nodules are composed of chondrocytes in groups. Variation in chondrocyte size is observed, but no signs of mitotic activity (Figure 4) [13].

Imaging findings in SC are very similar to those in other locations, such as large joints. Plain radiography shows calcified bony bodies, although calcification may not be seen in 5%-30% of cases. The CT shows a soft tissue mass with multiple calcifications and bony erosive phenomena. The most common MRI pattern is a mass with an intermediate signal on T1, a high signal on T2, and poor gadolinium enhancement. Malignant transformation is rare, but conversion to low-grade chondrosarcomas has been described [14].

Differential diagnoses include neurogenic tumors such as neurofibroma, schwannoma, meningioma, and, less frequently, malignant peripheral nerve sheath tumors, cystic tumors, synovial cysts, perineural cysts, chondroid neoplasms, and chondrosarcoma, including epidural abscesses. Chondromatosis can be easily confused with benign and malignant entities, such as tumor calcinosis, hamartoma, degenerative joint disease, extraskeletal chondroma, and chondrosarcoma.

Because it is a rare condition, much of the management has been inferred from extraspinal disease [4,7,15]. For patients with tumor spine pathology, mechanical instability, neurological symptoms, and refractory pain remain the best indications for surgery. Other indications for surgery include local tumor control and correction or prevention of deformity. However, there are many other factors that influence the surgical decision, such as patient comorbidities/status, tumor biology, RT/QT tumor sensitivity, spinal cord compression, etc. [15, 16].

In the case of SC, surgery is not always necessary for asymptomatic patients and with high surgical risk due to comorbidities, but the histopathology of the tumor should always be determined as there is a risk of malignant transformation with a 6-17% inferred recurrence of extraspinal disease. For symptomatic patients with low comorbidities, surgical treatment is preferred, consisting of resection of the free fragments, complete synovectomy, and partial or total relief of symptoms [7, 17].

In most cases of SC of the lumbar spine, total resection with extensive arthrodesis, including the sacroiliac joint, was performed. In our case, our team preferred a total resection with a minimally invasive approach to the spine in different stages, with the aim of preserving the patient's quality of life and desire to continue running. In our case, an LLIF was performed. Few cases have been reported with the use of these lateral approaches for the management of spinal tumors. In 2010, Uribe and colleagues published 21 cases of thoracic tumors with a lateral approach, where they reported improvements over the traditional open technique, with an average blood loss of 291 cc and a hospital stay of three days. Improvements of 62% in the Visual Analog Scale (VAS) and 53% in the Oswestry Disability Index (ODI) were noted. The only postoperative complication was pneumonia [18].

Regarding stand-alone LLIF vs. LLIF with fixation, the evidence is mixed and controversial. Chen et al. in 2019 [19] demonstrated that there was no major cage subsidence and demonstrated a satisfactory fusion rate at two years (86%).

A recent meta-analysis by Jiang et al. 2024, showed that patients with risk factors for subsidence benefited from posterior instrumentation [20]. The aim of performing LLIF was to subsequently perform total SC resection in relation to the right L3L4 facet. This procedure is associated with instability in axial rotation and lumbar flexion [21].

The use of minimally invasive spinal techniques facilitates post-surgical recovery and reduces the risk of complications and post-surgical systemic stress [16, 19, 22].

## Conclusions

We present the rare condition of lumbar SC managed with minimally invasive spine techniques in different stages. In recent years, great advances have been made regarding the management of spinal tumors (e.g., minimally invasive technique); however, the overall management of these patients remains in a constant state of flux. Optimal treatment requires a multidisciplinary approach with surgeons, radiologists, pathologists, and oncologists.

A minimally invasive, multi-stage approach to this large tumor pathology transforms a major surgery, with its risks and associated results, into three minimally invasive surgeries with fewer risks, short hospital stays, and good clinical and anatomical results.
